# CLE14 peptide delays broccoli senescence by regulating chlorophyll metabolism and reactive oxygen species homeostasis

**DOI:** 10.1186/s12870-025-07326-8

**Published:** 2025-10-14

**Authors:** Qiaomei Ma, Yuxiang Hu, Yumiao Xiao, Xingtong Song, Jiamiao Wu, Xiequan Ye, Zhenqing Zhao

**Affiliations:** Institute of Vegetables, Zhejiang Academy of Agricultural Sciences, No. 198 Shiqiao Road, Shangcheng District, Hangzhou, 310021 China

**Keywords:** Peptide, CLE, Broccoli senescence, Chlorophyll metabolism, Reactive oxygen species

## Abstract

CLE (CLAVATA3/Endosperm surrounding region-related) is a peptide hormone with multiple biological functions in plants. Previous studies have demonstrated that it plays an important role in modulating leaf senescence in *Arabidopsis*. However, the role of the CLE in broccoli senescence remains largely unknown. Here, we found that the expression of *BoCLE14* was highly induced in broccoli during storage, and exogenous application of 50 µM CLE14 significantly delayed the senescence of broccoli. Transcriptomic analysis revealed that chlorophyll metabolism and peroxisomes are involved in CLE14-mediated broccoli senescence. qRT-PCR analysis showed that the expression of the chlorophyll degradation genes *BoPPH*, *BoPAO*,* BoNYC1* and *BoCLH2* were inhibited by CLE14 treatment, resulting in higher levels of chlorophyll. Moreover, CLE14 treatment also prevented reactive oxygen species (ROS) accumulation by increasing antioxidant enzyme activities and the O_2_^•−^ scavenging rate. Overall, these results demonstrate that CLE14 treatment delays broccoli senescence by inhibiting chlorophyll degradation and improving the antioxidant capacity of broccoli, which provides both theoretical foundations and practical strategies for developing bioactive peptide-based green preservation technologies.

**Supplementary Information:**

The online version contains supplementary material available at 10.1186/s12870-025-07326-8.

## Introduction

Broccoli (*Brassica oleracea* L. var. italica) is one of the foremost vegetable crops globally known for its health-promoting bioactive components, including Vitamin C, proteins, phenols, flavonoids, and glucosinolates [1, 2]. However, prematurely harvested broccoli is prone to senescence owing to rapid metabolism, accompanied by yellowing and deterioration of nutritional quality [3]. Therefore, it is of great practical significance to explore preservation techniques to prolong the shelf life and maintain the quality of broccoli.

The senescence of post-harvest broccoli is strictly regulated by multiple phytohormones [4–6]. For instance, ethylene (ETH) plays a positive role in broccoli senescence; treatment with ethylene accelerates broccoli senescence [7], whereas 1-methylcyclopropene (1-MCP) treatment significantly delays broccoli yellowing [8]. Fang et al. [4] found that jasmonic acid (JA) also positively regulates broccoli senescence by promoting chlorophyll degradation. In contrast, brassinosteroids (BR), melatonin (MT), and cytokinins delay broccoli senescence by improving its antioxidant capacity and suppressing carotenoid metabolism and chlorophyll degradation [3, 5, 6]. Recently, several studies have found that plant peptides derived from precursors play greater-than-expected roles in plant signaling [9–11]. Peptides are a class of hormonal molecules [12]. Although extensive studies have revealed the importance of traditional hormones in broccoli senescence, the potential role of peptide hormones in this process remains unclear.

The CLAVATA3/Endosperm surrounding region-related (CLE) peptide family is a well-studied plant peptide family. *CLE* genes play critical roles in modulating diverse developmental and physiological processes, including stem cell homeostasis, vascular formation, lateral root establishment, seed development, and stress responses [13–15]. CLV3 is a short-range signaling molecule that transmits shoot apical meristem cell fate information to *Arabidopsis* [16]. Etchells and Turner [17] reported that CLE41 modulates the division of vascular cells and that overexpression of *CLE41* leads to a dramatic loss of ordered vascular tissue development. Araya et al. [18] revealed that the expression of *CLE3* is highly induced under N-deficient conditions to suppress the length and number of lateral roots, thereby suppressing root expansion in a low-nutrient environment in *Arabidopsis*. Takahash et al. [19] demonstrated that CLE25 modulates stomatal control during long-distance signaling. The expression of *CLE25* was highly induced in the roots by drought stress, and the CLE25 peptide was then transported to the leaves, triggering stomatal closure by enhancing the accumulation of abscisic acid. Recently, CLE peptides have been shown to participate in leaf senescence. CLE42 delayed leaf senescence by antagonizing the ETH pathway [20], and CLE14 acts as a “brake signal” to delay both age and stress induced leaf senescence by enhancing ROS scavenging capacity in *Arabidopsis* [21]. However, research on whether CLE regulates post-harvest vegetable crop senescence is lacking.

Based on preliminary experimentation, we found that the expression of *BoCLE14* was highly induced during storage, indicating that CLE14 may be involved in broccoli senescence. In the present study, an exogenous CLE14 peptide was used to explore the potential role of CLE14 in broccoli senescence. RNA-seq analysis was used to explore the possible biological mechanisms. This study revealed the role of peptide hormones in broccoli senescence and sheds new light on the preservation of broccoli.

## Materials and methods

### Plant materials and treatments

Broccoli (*Brassica oleracea* L. var. *Italica*) was collected from Shulan Agricultueal Farm in Hangzhou, Zhejiang Province. ‘Naihanyouxiu’ broccoli cultivar was used as plant material in the study, which was provided from Sakata Seed Corporation. And the broccoli was harvested at commercial maturity, characterized by tightly closed florets and a deep green color. For this experiment, broccoli of the same size, consistent color, and no mechanical damage were selected.

The CLE14 peptide was synthesized by Dongheng Biomedical Co., Ltd., China. A total of 300 broccoli heads were randomly assigned to 5 groups containing 60 broccoli heads each (three replicates, 20 broccoli heads per replicate) for the experimental treatments, and sprayed with 0 (control), 10, 50, 100 or 300 µM CLE14, respectively. After being dried overnight, the treated broccoli was packaged and stored at 4 ± 0.5℃ for 28 days. The appearance attributes were evaluated every 7 days. After each evaluation, the florets from the five broccoli groups were collected for subsequent gene expression and biochemical analyses.

### Broccoli yellowing index and color detection

The broccoli yellowing index (%) was used to assess the yellowing area of broccoli surfaces using the scale described by Luo et al. [22]. The color parameters of broccoli were valued using a digital colorimeter (CR-400, Konica Minolta, Japan). The values of lightness (L^*^) and hue angle (H^*^) were measured at five points on each broccoli every 7 days.

### Measurement of nutritional quality of broccoli

The soluble sugar content of broccoli was determined using a commercial assay kit (BC0030, Solarbio, China).

The soluble protein content in broccoli was measured using a BCA protein assay kit (ml095490, Shanghai Enzyme-linked Biotechnology, China). The intracellular proteins in broccoli were extracted using PBS buffer (50 mM, pH 7.4), and the soluble protein content was calculated by measuring the absorbance at 760 nm using BSA as a standard.

Broccoli endogenous Vitamin C content was detected using the solid blue salt colorimetric method descripted by Zhang and Huang [23]. Vitamin C content was calculated by measuring the absorbance at 420 nm.

Glucosinolate levels were quantified using a glucosinolate assay kit (ml092776, Shanghai Enzyme-Linked Biotechnology). Total glucosinolate content was determined by measuring the absorbance at 505 nm.

The phenolic and flavonoid contents were measured using the Folin-Ciocalteu method and NaNO_2_-Al(NO_3_)_3_-NaOH colorimetry method, respectively. First, broccoli samples (0.5 g) were mixed with 5 mL of methanol (80%) and subjected to ultrasonic treatment for 30 min. Supernatants were then collected for analysis. For phenolic content measurement, 200 µL of the supernatant was combined with 1 mL Folin-Ciocalteau and 800 µL Na_2_CO_3_ solution (75 g L^−1^), and the mixture was incubated at 25℃ for 1 h. The absorbance was then measured at 765 nm. For the total flavonoid content test, 1 mL of the supernatant was mixed with 1 mL 70% ethanol and 0.3 mL of 5% NaNO_2_. After mixing, 0.3 mL 10% Al(NO_3_)_3_ was added and the mixture was left to stand for 3 min at 25℃, 1 mL NaOH (1 mol L^−1^) was then added. The reaction mixture was detected at 510 nm after 10 min using rutin as a standard.

### Chlorophyll content

The chlorophyll content was assessed following the method described by Xu et al. [24]. Approximately 100 mg of broccoli powder was added to 5 mL of acetone/ethanol (2:1) solution. Chlorophyll content was analyzed based on absorbance at 664 nm and 645 nm.

### RNA extraction and quantitative PCR analysis

Total RNA was extracted from broccoli florets using an RNA Simple Total RNA Kit (Tiangen, China) with DNase I treatment. A ReverTra Ace quantitative (qPCR) reverse transcription (RT) kit (Toyobo, Japan) was used to synthesize cDNA. qRT-PCR assays were performed using the StepOne detection system (Thermo Fisher Scientific, USA) and SYBR Green PCR Master Mix Kit (Takara, Japan). Gene expression levels were calculated using the comparative ΔΔCt method. Briefly, the average Ct value for the target gene was normalized to the average Ct value of the endogenous reference gene (*BoActin*) to obtain the ΔCt value for each sample: ΔCt = Ct (target gene) - Ct (reference gene). The ΔΔCt value was then calculated as: ΔΔCt = ΔCt (test sample) - ΔCt (untreated sample). The relative fold change in gene expression was determined as 2^(-ΔΔCt). Three independent biological replicates were analyzed per experimental group. Sequences of primers are listed in Table S1.

### RNA-seq analysis

Three independent repeats from the control and CLE14-treated broccoli at 0, 7, 14, 21, and 28 d post-harvest were used for RNA-seq analysis. RNA was extracted using the RNAprep Pure Plant Kit (Tiangen, China) and quantified using a NanoDrop 2000 spectrophotometer (Thermo Fisher Scientific). RNA integrity was determined using the RNA Nano 6000 Assay Kit of the Agilent Bioanalyzer 2100 system. Biomarker Technologies (Beijing, China) prepared Illumina libraries and sequenced them on an Illumina NovaSeq 6000 sequence platform (150 bases paired-end reads; Illumina, USA). The raw reads were further processed using the bioinformatics pipeline tool, and the BMKCloud (www.biocloud.net) online platform. Raw reads in fastq format were first processed through in-house Perl scripts, and the reads containing adapter or poly-N and low-quality reads were removed to obtain clean reads for further analyses. The proportion of clean reads in the samples (Q30) ranged from 94.62 to 97.82%. The cleaned reads were aligned to the *Brassica oleracea* reference genome from NCBI using hisat2 tools soft with default parameters. Gene expression levels were quantified by mapping fragments per kilobase of transcript per million fragments mapped (FPKM). Additional, differential expression analysis between treatments were performed using the DESeq2 package, and the genes with an adjusted *P*-value < 0.01 & Fold Change ≥ 2 were assigned as differentially expressed. Randomly chosen deferentially expressed genes were verified using qRT-PCR. The weighted gene co-expression network analysis (WGCNA) (min Module Size 30, and merge Cut Height, 0.25) was used to build a co-expression network. Data were analyzed using the online BMKCloud bioinformatics platform.

### Malondialdehyde (MDA), H_2_O_2_ content and O_2_^•−^ production rate

The MDA levels were measured following Xu et al. [24]. Broccoli floret powder was mixed with 5 mL trichloroacetic acid, and the supernatant was collected via centrifugation. The supernatant was combined with 2 mL of thiobarbituric acid (0.67%) and boiled for 20 min. The MDA content was analyzed by measuring the absorbance at 450, 532, and 600 nm.

H_2_O_2_ content was measured using a Micro Hydrogen Peroxide (H_2_O_2_) Assay Kit (BC3590, Solarbio, China).

The generation rate of superoxide anions (O_2_^•−^) was determined using hydroxylamine oxidization according to Shi et al. [25]. Briefly, broccoli (0.5 g) was mixed with 2 mL of PBS buffer, and the supernatant was collected. The supernatant was mixed with 100 µL NH_2_OH·HCl (1 mM), 1 mL PBS buffer and subjected to incubation at 25℃ for 1 h. Following this, 1 mL C_6_H_7_NO_3_S (17 mM) and 1 mL C_10_H_7_NH_2_ (7 mM) were added. The generation rate of O_2_^•−^ was calculated based on absorbance at 530 nm after incubation at 25℃ for 20 min, using NaNO_2_ as the standard.

### Enzyme activities

The activities of pheophorbide a oxygenase (PAO) and pheophytinase (PPH) were measured using Plant enzyme activity Kits (Enzyme-linked Biotechnology).

Catalase (CAT) and superoxide dismutase (SOD) activity was measured as described by Cheng et al. [26]. Broccoli floret powder (0.5 g) was added to 3 mL of PBS buffer (50 mM, pH 7.8, with 0.2 mM EDTA, 2mM AsA, 25 mM HEPES, and 2% polyvinylpolypyrrolidone [w/v]). The supernatant was then collected for enzyme activity assay, which was performed using a SHIMADZU UV-2600 spectrophotometer (Shimadzu, Kyoto, Japan).

### Statistical analysis

All data were analyzed using Statistical Analysis System version 8 (SAS Institute Inc., Cary, NC, USA). The significance of treatment differences was analyzed using ANOVA followed by Tukey’s test at the 5% significance level. Data were recorded as the mean ± standard errors (SE) of at least three independent biological replicates.

## Results

### Effects of exogenous CLE14 treatment on the appearance attributes of broccoli

**Fig. 1:**
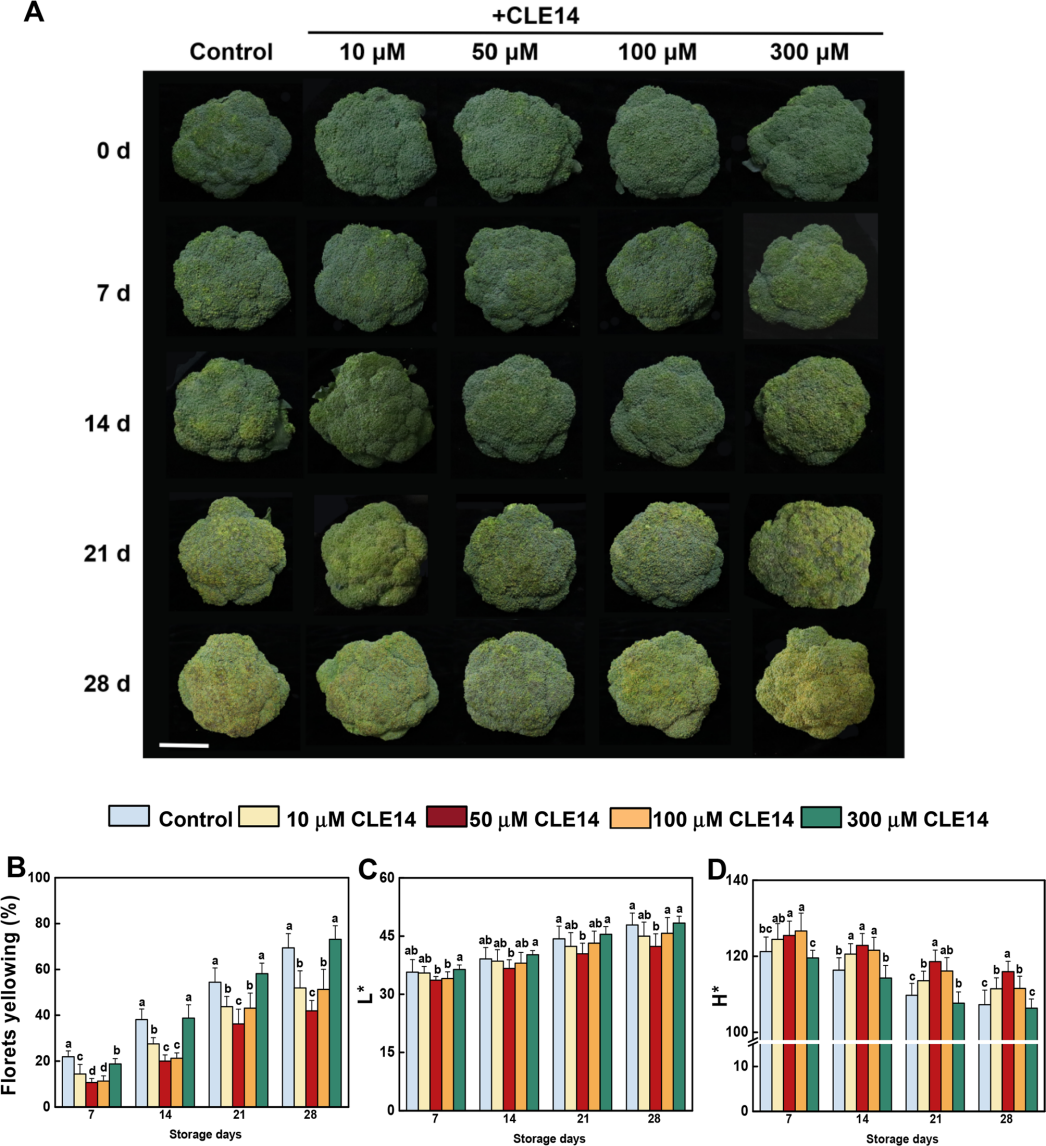
Effects of exogenous CLE14 treatment on the appearance attributes of broccoli. (**A**) Changes in phenotype of control and CLE14-treated broccoli during storage. Scale bar = 5 cm. (**B**) Yellowing index, (**C**) lightness value (L^*^) and (**D**) hue angle (H^*^)of broccoli florets. Different letters indicate significant differences among treatments on the same day (*p* < 0.05, Tukey’s test)

To explore the potential role of the CLE peptides in broccoli senescence, changes in the expression of CLE biosynthesis genes after broccoli harvest were detected using RT-qPCR. Notably, the expression of *BoCLE14*, which is highly homologous to AtCLE14 in *Arabidopsis* (Fig. S1), was upregulated and reached extremely high levels after 14 days (Fig. S2), indicating that CLE14 was involved in broccoli senescence. Exogenous CLE14 was used to treat post-harvest broccoli, and appearance attributes were measured. The results showed that application of CLE14 at 10, 50, 100 µM significantly delayed the yellowing of broccoli during storage, and broccoli treated with 50 µM CLE14 displayed the lowest yellowing index (Fig. 1A and B). However, 300 µM CLE14 treatment had no effect on the yellowing of broccoli. The L* value of broccoli also showed a rising trend during storage; however, CLE14 treatment significantly suppressed this increase. In contrast to the lower L* value, CLE14-treated broccoli exhibited higher H* values than that of the control, exce*p*t for in the case of the 300 µM CLE14-treatment (Fig. 1C and D).

### Effects of CLE14 on the nutritional quality of broccoli

**Fig. 2:**
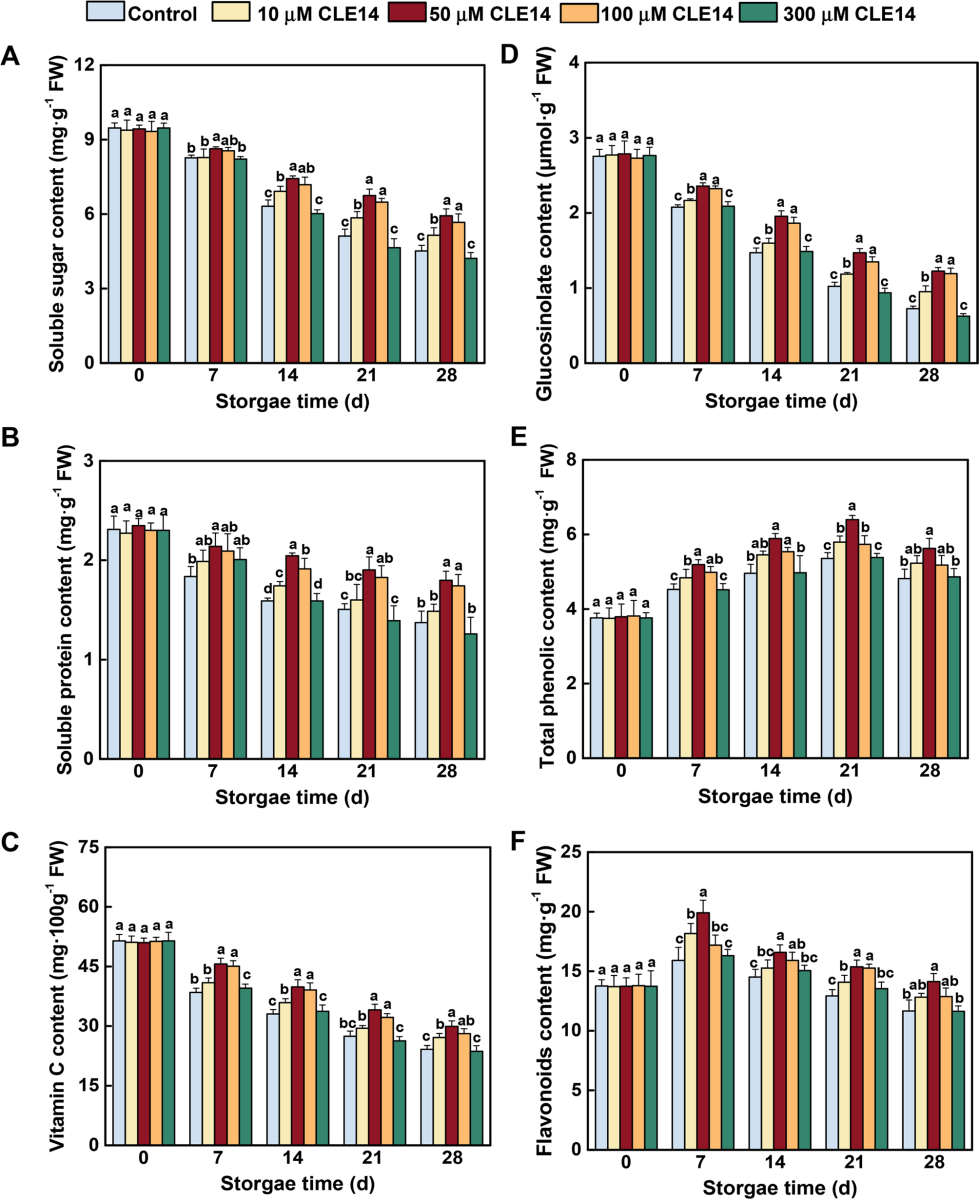
Effect of treatments with different concentrations of exogenous CLE14 on the nutritional quality of broccoli, including (**A**) soluble sugar, (**B**) soluble protein, (**C**) Vitamin C, (**D**) total glucosinolate, (**E**) total phenolic, and (**F**) flavonoids during storage period from 0 d to 28 d. Data are presented as the means ± SD of three biological replicates (*n* = 3). Different letters indicate significant differences among treatments on the same day (*p* < 0.05, Tukey’s test)

The effects of exogenous CLE14 treatment on the nutritional quality of broccoli were evaluated. During storage, the contents of soluble protein, sugar, Vitamin C and glucosinolate showed a continuous downward trend, however, CLE14-treated broccoli displayed a slower rate of decline (Fig. 2), especially in broccoli treated with 50 µM and 100 µM CLE14. In contrast, the phenolic and flavonoid contents initially increased and then decreased. CLE14 treatment significantly increased the accumulation of total phenolic and flavonoids compared to that in the control. Similar to the appearance attributes, 300 µM CLE14 treatment didn’t affect the decrease in quality of broccoli during storage.

### RNA-seq analysis revealed biological processes associated with CLE14-regulated broccoli senescence

**Fig. 3:**
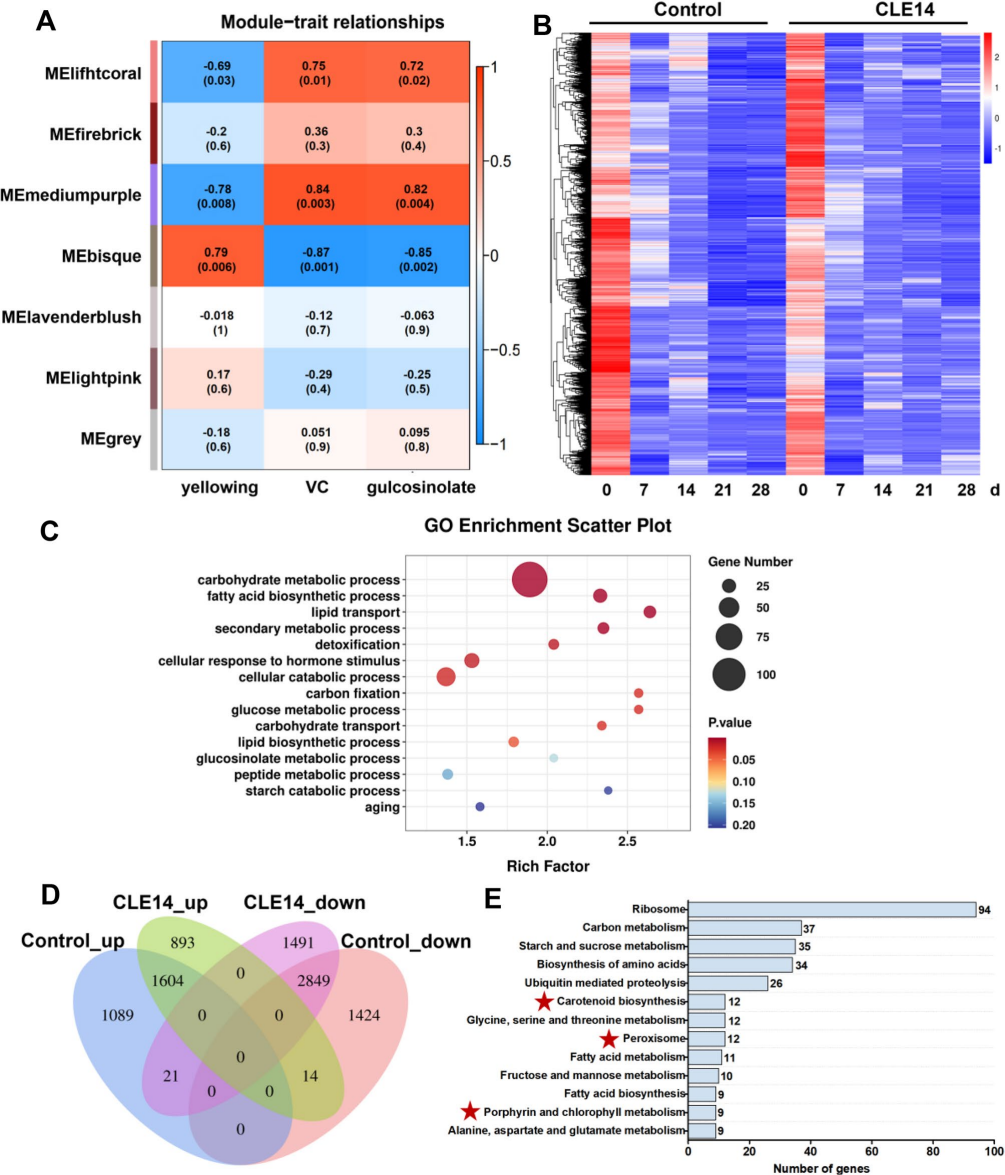
RNA-seq analysis revealed biological processes correlated to CLE14-regulated broccoli senescence. (**A**) Module-trait relationships between modules and yellowing index, Vitamin C, and glucosinolate content. Each row represents a module characteristic gene, each cell shows the corresponding correlation coefficient and *p* value. (**B**) Heat map depicts the transcription level of genes in the mediumpurple module of control and CLE14-treated broccoli at different senescence stages. (**C**) Enriched GO biological process of mediumpurple module genes (Table S3). (**D**) Venn diagram shows the overlap between up-regulated or down-regulated genes at 7 day compared to 0 day in control and CLE14-treated broccoli, respectively. (**E**) KEGG pathway analysis of 2384 genes with inconsistent expression pattern in control and CLE14-treated broccoli from 0 d to 7 d

To gain a deeper understanding of the changes during the senescence process of broccoli, RNA-seq analysis of the control and 50 µM CLE14 treatments were performed. We examined the co-expressed gene clusters (modules) that were highly correlated with CLE14-delayed senescence in broccoli using WGCNA. As a result, 53,649 genes were separated into seven modules (Fig. 3A). The medium-purple module showed the most significant positive correlation with Vitamin C and glucosinolate, and the most significant negative correlation with the yellowing index, indicating that the genes in the medium-purple module were highly correlated with CLE14-delayed senescence. The heat map of the multiple expression changes of 4736 genes in the medium-purple modules indicated a slower decline in expression levels in CLE14-treated broccoli than in the control (Fig. 3B, Table S2). The Gene Ontology (GO) enrichment analysis revealed that CLE14 treatment affected biological processes related to carbohydrate metabolism, fatty acid biosynthesis, lipid transport and biosynthesis, detoxification, hormone response, cellular catabolism, glucosinolate metabolism, peptide metabolism, and aging (Fig. 3C, Table S3).

Furthermore, senescence-associated genes differently regulated between control and CLE14 treatment were identified (fold change ≥ 2 and *P* < 0.05) between 0 d and 7 d, given that CLE14 treatment is closely associated with the onset of broccoli senescence. There were 2714 upregulated and 4287 downregulated genes in the control, whereas 2511 genes were upregulated and 4361 genes were downregulated in the CLE14 treatment from 0 to 7 d (Fig. 3D, Table S4). Among the senescence-associated genes, the expression of 2384 genes was explicitly altered in the CLE14 treatment compared to that in the control (Table S5). Kyoto Encyclopedia of Genes and Genomes (KEGG) analysis showed that CLE14-mediated broccoli senescence was highly associated with diverse metabolic pathways involving carbon, amino acids, starch, sucrose, fatty acids, glycine, serine, and threonine (Fig. 3E, Table S6). In addition, the expression levels of genes correlated with carotenoid, porphyrin, and chlorophyll metabolism, and peroxisomes, two major regulators of senescence, were also altered in CLE14-treated broccoli, indicating that CLE14 treatment-delayed broccoli senescence may be related to these two pathways.

### CLE14 treatment inhibits chlorophyll degradation

**Fig. 4:**
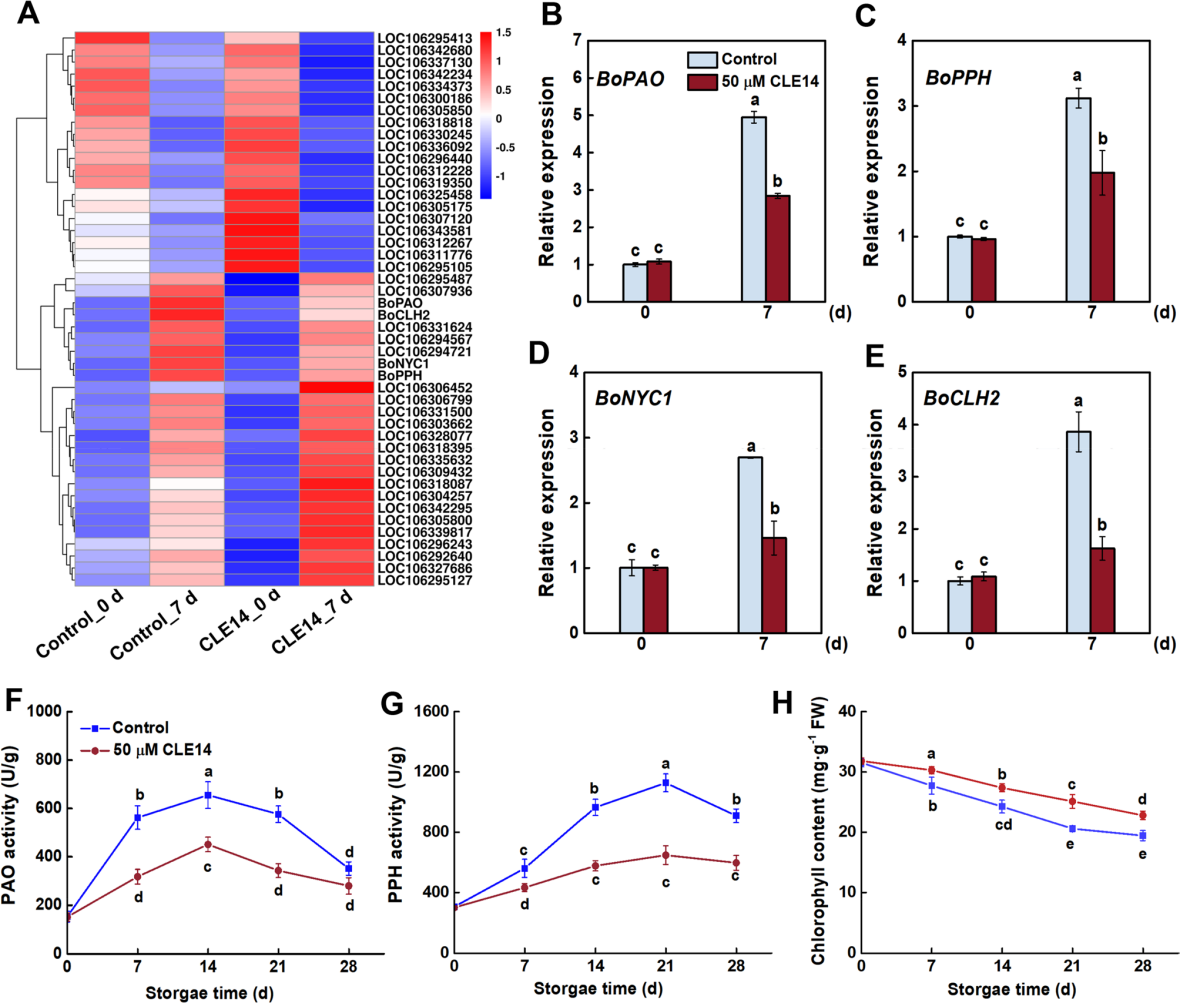
Effect of exogenous CLE14 treatment on the chlorophyll metabolism of broccoli. (**A**) Heat map depicts the transcription level of genes involved in carotenoid and porphyrin & chlorophyll metabolism obtained by RNA-seq. (**B-E**) Selected genes were assessed by qRT-PCR and *BoActin* was used as internal reference. (**F-G**) Activities of PPH and PAO in control and CLE14-treated during storage. (**H**) The chlorophyll contents content in control and CLE14-treated broccoli at different senescence stages. Data in (**B-H**) are presented as the means ± SD of three biological replicates (*n* = 3). Different letters indicate significant differences among treatments on the same day (*p* < 0.05, Tukey’s test)

Chlorophyll degradation is a key hallmark of broccoli senescence and directly facilitates visual yellowing during postharvest deterioration. RNA-seq analysis showed that carotenoid, porphyrin, and chlorophyll metabolism were significantly altered by CLE14 treatment. As shown in Fig. 4A, genes involved in chlorophyll degradation, such as pheophorbide a oxygenase (*BoPAO*), chlorophyllase (*BoCLH2*), non-yellow coloring 1 (*BoNYC1*), and pheophytinase (*BoPPH*), were highly upregulated at day 7 in the control group. However, the senescence-induced upregulation of these genes was significantly inhibited by CLE14 treatment. qRT-PCR was performed for validation, and the results showed that the expression levels of those genes in CLE14-treated broccoli were significantly lower than that in the control, which was consistent with the RNA-seq results (Fig. 4B–E). In addition, changes in PAO and PPH activity and chlorophyll content in broccoli at different senescence stages were assayed. The PPH and PAO activity first increased and then decreased in both CLE14-treated broccoli and the control, whereas the PPH and PAO activity in the CLE14-treated broccoli were lower than those in the control (Fig. 4F–G). Moreover, chlorophyll levels showed a notable downward trend during storage, and chlorophyll degradation was significantly suppressed by the application of CLE14 (Fig. 4H).

### CLE14 treatment enhances ROS homeostasis

**Fig. 5:**
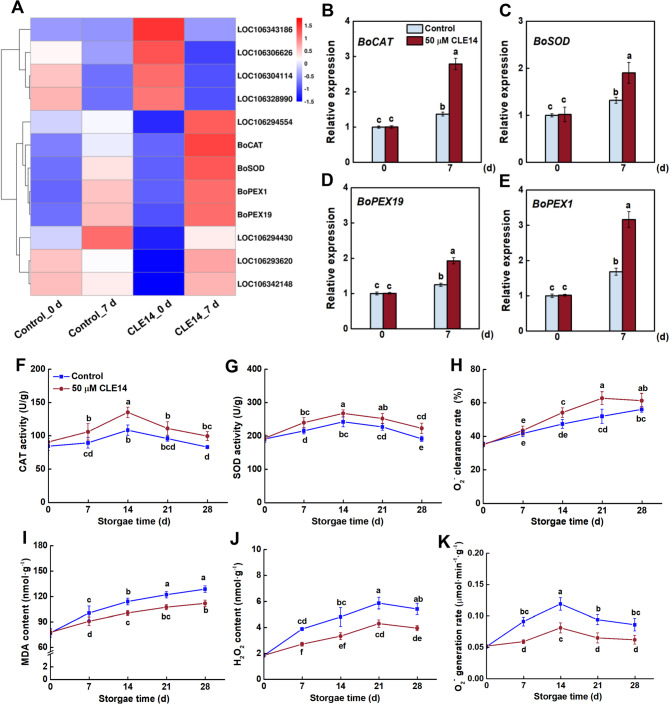
Effect of exogenous CLE14 treatment on the peroxisome. (**A**) Heat map depicts the transcription level of genes involved in peroxisome obtained by RNA-seq. (**B-E**) Selected genes were assessed by qRT-PCR, and *BoActin *was used as internal reference. Activities of CAT (**F**), SOD (**G**) and the O_2_^•−^clearance rate (**H**) in control and CLE14-treated during storage. Effect of exogenous CLE14 treatment on the accumulation of MDA (**I**), H_2_O_2_(**J**), and the generation rate of O_2_^•−^(**K**) in control and CLE14-treated broccoli at different senescence stages. Data in (**B-K)** are presented as the means ± SD of three biological replicates (*n* = 3). Different letters indicate significant differences among treatments on the same day (*p* < 0.05, Tukey’s test)

Peroxisomes are single-layer membrane-coated organelles that act as both sources and regulators of ROS, and play an important role in plant senescence. In this study, the peroxisome process was significantly altered by CLE14 treatment. The transcription levels of the *peroxisome biosgenesis factor 1* (*BoPEX1*) and *peroxisome biosgenesis factor 19* (*BoPEX19*), as well as the key antioxidant genes *catalase* (*BoCAT*) and *superoxide dismutase gene* (*BoSOD*) were notably upregulated by CLE14 treatment (Fig. 5A). The changes in the expression patterns of these genes were validated by qRT-PCR analysis (Fig. 5B–E), indicating that CLE14 treatment may delay broccoli senescence by modulating the ROS scavenging capacity. Therefore, the antioxidant enzyme activity of broccoli was determined. The CAT and SOD activity first increased and then decreased, reaching a peak at 7 and 14 d, respectively (Fig. 5F–G). Notably, CLE14 treatment dramatically improved CAT and SOD activity over the entire storage period. On the contrary, the scavenging rate of O_2_^•−^ displayed a continuous upward trend during storage, and it was also accelerated with CLE14 treatment (Fig. 5H). We also assayed the effect of CLE14 treatment on ROS accumulation in broccoli. The MDA content displayed an overall increasing trend in both control and CLE14-treated broccoli, but the increase was greatly suppressed by CLE14 treatment (Fig. 5I). Consistent with the lower MDA contents, CLE14-treated broccoli also displayed lower accumulation of H_2_O_2_ and a lower O_2_^•−^ generation rate compared to that of the control (Fig. 5J–K). Together, these results demonstrate that the exogenous application of CLE14 can efficiently inhibit ROS accumulation in broccoli during storage.

## Discussion

The CLE peptide family is among the most well-known plant peptide families. CLE peptides participate in the regulation of shoot meristem maintenance, vascular development, root growth, leaf senescence, and stress response [18, 19, 27–29]. However, the putative role of CLE in modulating post-harvest vegetable crop senescence and its underlying mechanisms remain unknown. Here, we revealed that CLE14 delays broccoli senescence by inhibiting chlorophyll degradation and promoting ROS homeostasis.

Our study showed that the transcription level of *BoCLE14* in stored broccoli was highly induced (Fig. S2). Moreover, exogenous application of a low concentration of the CLE14 peptide delayed broccoli senescence, including better appearance attributes and higher nutritional quality. Conversely, treatment with a high concentration of CLE14 peptide accelerated the senescence of broccoli, suggesting that the CLE14 peptide functions as a hormone-like signaling molecule (Figs. 1 and 2). Based on these results, we found that CLE14 functions as a negative regulator in the postharvest senescence of broccoli. However, this conclusion remains to be substantiated by genetic evidence, primarily due to the underdeveloped genetic transformation system in broccoli. Combined with previous reports, CLE14 and CLE42 treatments delayed leaf senescence in *Arabidopsis*, indicating that CLE can modulate both age-dependent and postharvest senescence in plants. Similarly, the phytosulfokine (PSK) peptide is involved in regulating both age-dependent and post-harvest senescence in plants. PSRK1 is the receptor for the PSK peptide, and Matsubayashi et al. [30] found that *pskr1-1 mutants* exhibit premature leaf senescence in *Arabidopsis*. In addition, exogenous PSK treatment markedly delays the senescence of broccoli, strawberries, and loquats during cold storage [31–33].

Transcriptomic profiling suggested that CLE14-dependent senescence-regulated genes were highly associated with chlorophyll metabolism and peroxisomes, which was validated using qRT-PCR analysis (Fig. 3). Yellowing is the most obvious sign of broccoli senescence and is mainly due to chlorophyll degradation [22, 34]. According to previous studies, chlorophyll degradation is regulated by various genes, including *PAO*, *PPH*,* NYC1* and *CLH2* [35–37]. Our results revealed that CLE14 treatment downregulated the expression of the chlorophyll degradation genes *BoPPH*, *BoPAO*,* BoNYC1* and *BoCLH2*, and that PAO and PPH activity was greatly suppressed in CLE14-treated broccoli during storage (Fig. 4). Moreover, our results revealed that exogenous treatment with CLE14 greatly alleviated the decrease in chlorophyll in broccoli (Fig. 4), which is consistent with the results of a previous study in which CLE42 treatment delayed the age-dependent and dark-induced decline in chlorophyll in *Arabidopsis* [20]. Notably, several studies have suggested that inhibiting the gene expression or activities involved in chlorophyll degradation can efficaciously delay the yellowing of broccoli. For instance, folic acid treatment significantly suppressed the expression of *BoPPH*, *BoPAO* and *BoNYC1* and delayed broccoli yellowing [24]. Wu et al. [38] reported that melatonin-treated broccoli exhibited delayed senescence, accompanied by a reduction in the expression of chlorophyll catabolism genes. In addition, treatment with l-phenylalanine significantly delays broccoli yellowing by downregulating the activities of PPH, PAO, and CLH [39]. In summary, these findings suggest that CLE14 plays a vital role in preventing chlorophyll degradation and delaying broccoli senescence by inhibiting the activity of chlorophyll-degrading enzymes.

Reactive oxygen species (ROS), like O_2_^•−^, and H_2_O_2_, are specific molecular regulators for cell signaling and function [40–42]. However, the overproduction of ROS is toxic to plant cells, is the primary cause of membrane degradation, and ultimately accelerates senescence [43–45]. A complex antioxidant system has evolved, which is mostly regulated by ROS-scavenging enzymes, such as CAT, SOD, POD, and APX [46, 47]. Peroxisomes have been demonstrated to have ROS-mediated functions in oxidative reactions that are characteristic of senescence [48, 49]. In the current study, the expression levels of *BoPEX1*, *BoPEX19*, *BoCAT* and *BoSOD* were notably upregulated by CLE14 treatment, and CAT and SOD activity significantly increased in CLE14-treated broccoli during storage, consistent with the reduction in ROS accumulation in CLE14-treated broccoli (Fig. 5). In agreement with our findings, the overexpression of *CLE14* improved the transcription levels of ROS-scavenging genes, and lower levels of ROS accumulation in the leaves were detected in *Arabidopsis* [21]. While, CLE42 and CLE41/44 mediate leaf senescence by antagonizing the ethylene pathway [20]. Given that ethylene signaling is known to regulate ROS homeostasis, which plays a an important role in senescence [50], the changes in ROS levels observed under pCLE14 treatment may be an indirect effect of suppressing ethylene signaling. Future studies of the relationship between CLE14 and ethylene pathway could help disentangle these effects. In addition, multiple plant hormones have been shown to modulate broccoli senescence [3, 5, 6]. It would be interesting to elucidate whether CLE14 interacts with ethylene or other plant hormones to modulate post-harvest senescence.

## Conclusion

This study demonstrates that peptide hormone CLE14, especially at 50 µM, has a positive effect on delaying senescence and maintaining high quality in broccoli during storage. Transcriptomic profiling revealed that the lower expression of the chlorophyll degradation genes *BoPPH*, *BoPAO*,* BoNYC1* and *BoCLH2* may explain the higher chlorophyll levels in CLE14-treated broccoli. In addition, the accumulation of ROS was highly suppressed by the application of CLE14, which is consistent with the enhanced ROS-scavenging capacity of broccoli. This study expands our knowledge of the functions of peptide hormones in post-harvest vegetable crop senescence and sheds light on vegetable crop preservation.

## Supplementary Information

Supplementary Material 1. Table S1 Primers used in this study. Table S2 Transcript levels of mediumpurple module genes in broccoli treated with CLE14 and the control. Table S3 Gene Ontology analysis of genes in the mediumpurple module. Table S4 The differentially expressed genes between control and CLE14-treated broccoli within 0 to 7 d. Table S5 Genes with inconsistent expression patterns between control and CLE14-treated broccoli within 0 to 7 d. Table S6 KEGG pathways of 2384 CLE14-dependent senescence-induced genes.

Supplementary Material 2. Fig. S1 Amino acid sequence alignments of CLE. Fig. S2 Relative expression of *BoCLE *genes at different stages of broccoli senescence.

## Data Availability

Data will be available on request to corresponding authors. The sequencing data has been uploaded to the NCBI Sequence Read Archive (SRA) under BioProject accession PRJNA1306200. And the reference genome and gene model annotation files of Broccoli (Brassica oleracea L. var. botrytis L Planch) were downloaded from NCBI (GCA_900416815.2).

## Abbreviations

ROS: Reactive oxygen species
ETH: Ethylene
1-MCP: 1-methylcyclopropene
JA: Jasmonic acid
BR: Brassinosteroids
MT: Melatonin
CLE: CLAVATA3/Endosperm surrounding region-related
FPKM: Per kilobase of transcript per million fragments mapped reads
WGCNA: Weighted gene co-expression network analysis
MDA: Malondialdehyde
PAO: Pheophorbide a oxygenase
PPH: Pheophytinase
CAT: Catalase
SOD: Superoxide dismutase
GO: Gene ontology
KEGG: Kyoto Encyclopedia of Genes
NYC1: Genomesnon-yellow coloring 1
PEX: Peroxisome biosgenesis factor
PSK: Phytosulfokine
