# Supplementary material for: CLE14 peptide delays broccoli senescence by regulating chlorophyll metabolism and reactive oxygen species homeostasis
Source: BMC Plant Biol. 2025 Oct 14;25:1369. doi: 10.1186/s12870-025-07326-8 (PMC12522741; doi:10.1186/s12870-025-07326-8)
Supplement: Supplementary file 2 — Supplementary Material 2. Fig. S1 Amino acid sequence alignments of CLE. Fig. S2 Relative expression of BoCLE genes at different stages of broccoli senescence. [file 12870_2025_7326_MOESM2_ESM.doc]

**Sup****plemental Information:**

**Article title:** CLE14 peptide delays broccoli senescence via regulating chlorophyll metabolism and ROS homeostasis

**Qiaomei Ma^a^, Yuxiang Hu^a^, Yumiao Xiao^a^, Xingtong Song^a^, Jiamiao Wu^a^, Xiequan Ye^a^, Zhenqing Zhao^a*^**

**Fig. S1**

**A**

**B**


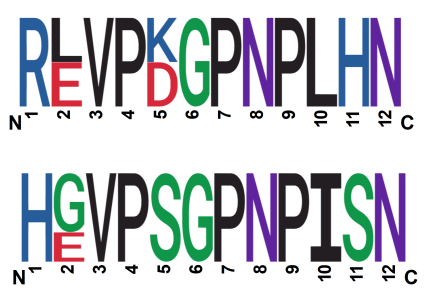


**AtCLE14**

**BoCLE14**

**AtCLE41**

**BoCLE41**

**AtCLE42**

**BoCLE42**


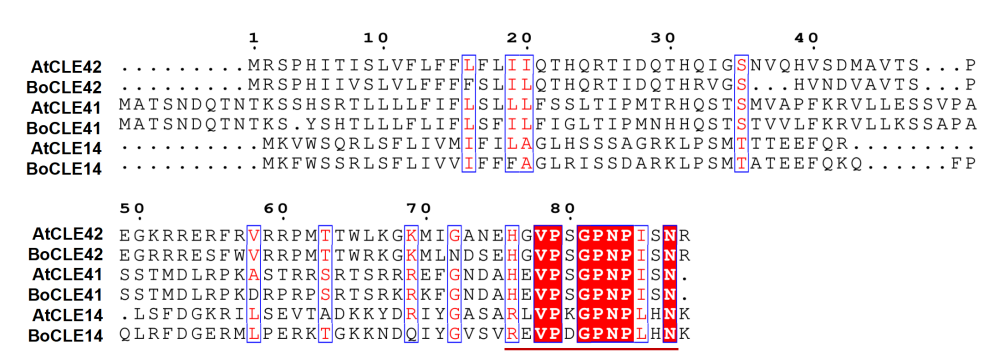


**Figure S1.** Amino acid sequence alignments of CLE14, CLE41 and CLE42 from Arabidopsis and broccoli using the Clustal X program (A). The sequences of 12 amino acid CLE motif was retrieved manually, and the sequences were plotted by Texshade (B).

**Fig. S2**


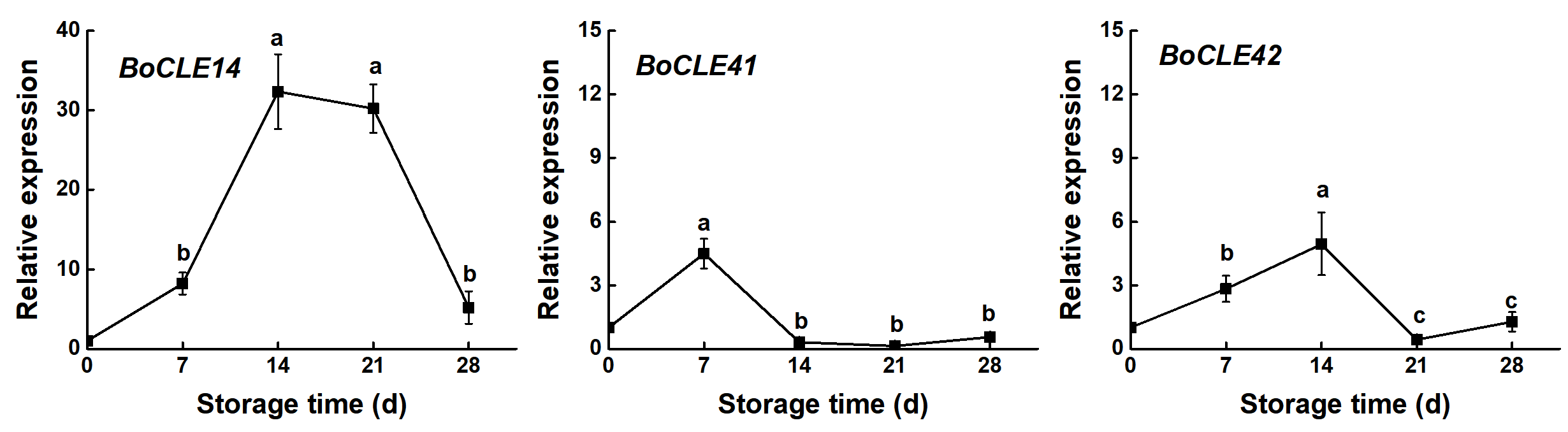


**A**

**B**

**C**

**Figure S2.** Relative expression of *BoCLE14*, *BoCLE41* and *BoCLE42* at different stages of broccoli senescence. *BoActin* was used as internal reference. Data are presented as the means ± SD of three biological replicates (n = 3).
